# Amodiaquine promotes testosterone production and de novo synthesis of cholesterol and triglycerides in Leydig cells

**DOI:** 10.1016/j.jlr.2021.100152

**Published:** 2021-11-19

**Authors:** Yujeong Choi, Eun Goo Lee, Gibbeum Lee, Mi Gyeong Jeong, Hyo Kyeong Kim, Ji-Hyun Oh, Sung Won Kwon, Eun Sook Hwang

**Affiliations:** 1 College of Pharmacy and Graduate School of Pharmaceutical Sciences, Ewha Womans University, Seoul, Korea; 2 Department of Pharmacy and College of Pharmacy, Seoul National University, Seoul, Korea

**Keywords:** amodiaquine, cholesterol synthesis, lipidomics, nuclear receptor 4A1, steroidogenesis, steroidogenic acute regulatory protein, CYP17A1, 3βHSD, CYP11A1, testosterone deficiency, 3β-HSD, 3β-hydroxysteroid dehydrogenase, ACC1, acetyl-CoA carboxylase 1, AQ, amodiaquine, CYP, cytochrome p450, CYP11A1, cytochome P450 family 11 subfamily A member 1, CYP17A1, cytochrome P450 family 17 subfamily A member 1, DGAT, diacylglycerol acyltransferase, GPAT, glycerol-3-phosphate acyltransferase, HEK293T, human embryonic kidney 293T cell line, HMGCR, hydroxy-methylglutaryl-CoA reductase, LH, luteinizing hormone, LHR, luteinizing hormone receptor, LPAAT, lysophosphatidic acid acyltransferase, NBRE, NR4A1-binding responsive element, NR4A1, nuclear receptor 4A1, PAP, phosphatidic acid phosphatase, PC, phosphatidylcholine, PE, phosphatidylethanolamine, PPARγ, peroxisome proliferator-activated receptor γ, SF-1, steroidogenic factor 1, StAR, steroidogenic acute regulatory protein, TG, triglyceride

## Abstract

Testosterone is a hormone essential for male reproductive function. It is produced primarily by Leydig cells in the testicle through activation of steroidogenic acute regulatory protein and a series of steroidogenic enzymes, including a cytochrome P450 side-chain cleavage enzyme (cytochome P450 family 11 subfamily A member 1), 17α-hydroxylase (cytochrome P450 family 17 subfamily A member 1), and 3β-hydroxysteroid dehydrogenase. These steroidogenic enzymes are mainly regulated at the transcriptional level, and their expression is increased by the nuclear receptor 4A1. However, the effect on Leydig cell function of a small molecule-activating ligand, amodiaquine (AQ), is unknown. We found that AQ effectively and significantly increased testosterone production in TM3 and primary Leydig cells through enhanced expression of steroidogenic acute regulatory protein, cytochome P450 family 11 subfamily A member 1, cytochrome P450 family 17 subfamily A member 1, and 3β-hydroxysteroid dehydrogenase. Concurrently, AQ dose-dependently increased the expression of 3-hydroxy-3-methylglutaryl-CoA reductase, a key enzyme in the cholesterol synthesis pathway, through induction of the transcriptional and DNA-binding activities of nuclear receptor 4A1, contributing to increased cholesterol synthesis in Leydig cells. Furthermore, AQ increased the expression of fatty acid synthase and diacylglycerol acyltransferase and potentiated de novo synthesis of fatty acids and triglycerides (TGs). Lipidomics profiling further confirmed a significant elevation of intracellular lipid and TG levels by AQ in Leydig cells. These results demonstrated that AQ effectively promotes testosterone production and de novo synthesis of cholesterol and TG in Leydig cells, indicating that AQ may be beneficial for treating patients with Leydig cell dysfunction and subsequent testosterone deficiency.

Steroids, known as hormones, have been identified as a broad range of molecules that are synthesized in the human body and have a variety of physiological effects. They have been used to treat a wide range of conditions, including asthma, chronic obstructive pulmonary disease, arthritis, inflammatory bowel disease, and multiple sclerosis, because of their anti-inflammatory and immune-modulating properties (1). The two major types of steroids are corticosteroids and sex steroids. Corticosteroids are typically produced by the adrenal cortex and are of two types: glucocorticoids and mineralocorticoids. Glucocorticoids, including corticosterone and cortisol, are important for regulating glucose and lipid metabolism in adipose tissue and for immune modulation to slow or stop the inflammatory response (2). Mineralocorticoids, such as aldosterone, promotes sodium reabsorption to maintain water balance (3). Sex steroids, including androgens, estrogens, and progestogens, are typically produced by the gonads or placenta and are important for the development of sexual characteristics. Natural steroid hormones are mainly synthesized in the gonads and adrenal glands through the signaling of the hypothalamic-pituitary axis (4). In particular, testosterone, a male sex hormone, is synthesized from cholesterol in the testicular Leydig cells by the activation of multiple steroidogenic enzymes in response to the sequential release of hypothalamic gonadotropin-releasing hormone and pituitary luteinizing hormone (LH) (5, 6, 7). Adequate testosterone production by interstitial Leydig cells is important for spermatogenesis and male fertility, and abnormally low testosterone causes symptoms, such as low energy, poor concentration, depression, and erectile dysfunction (8, 9). Testosterone deficiency results from the dysregulation of the hypothalamic-pituitary-gonadal axis and Leydig cell function and is also associated with chronic diseases, including diabetes, obesity, anemia, and infections (10, 11). Therefore, an understanding of steroidogenesis and its regulation is expected to be helpful in the treatment of testosterone deficiency.

LH or human chorionic gonadotropin stimulates testosterone biosynthesis in Leydig cells by binding to its receptor (luteinizing hormone receptor [LHR]) and increasing mitochondrial cholesterol transport through steroidogenic acute regulatory protein (StAR) (12, 13). Cholesterol is quickly converted into pregnenolone by the cytochome P450 family 11 subfamily A member 1 (CYP11A1) enzyme in the inner mitochondrial membrane and subsequently undergoes structural changes into testosterone by a series of enzymatic reactions catalyzed by 3β-hydroxysteroid dehydrogenase (3β-HSD), cytochrome P450 family 17 subfamily A member 1 (CYP17A1), and 17β-HSD in the endoplasmic reticulum (6). Leydig cells not only take cholesterol from the blood but also are able to synthesize cholesterol de novo to ensure steroidogenesis (14, 15). Cholesterol is synthesized from acetyl-CoA through the activation of a series of multiple enzymes, including hydroxy-methylglutaryl-CoA synthase and hydroxy-methylglutaryl-CoA reductase (HMGCR). Abundant acetyl-CoA is also converted into fatty acids through the action of acetyl-CoA carboxylase 1 (ACC1) and FASN and is utilized as a component of structural lipids or stored as triglycerides (TGs) (15, 16). Interestingly, steroidogenic enzymes are regulated at the transcriptional level through binding of nuclear receptor 4A1 (NR4A1), nuclear receptor 5A1 (also known as steroidogenic factor 1 [SF-1]), and peroxisome proliferator-activated receptor γ (PPARγ) to the gene promoter (17, 18, 19, 20). NR4A1-binding responsive elements (NBREs), steroidogenic factor-1-binding elements, and/or PPARγ-responsive elements are found to be within the promoters of many steroidogenic genes, and in the FASN and HMGCR gene promoters (21). Targeting the expression of LHR-independent steroidogenic enzymes may be a beneficial approach to modulate cholesterol or testosterone biosynthesis in Leydig cells.

Amodiaquine (AQ) has antimalarial and anti-inflammatory properties and is a potent NR4A1 ligand and a beneficial therapeutic for the treatment of neurodegenerative diseases (22). Although AQ plays an important role in immune modulation, little is known about its effect on the steroidogenic function of Leydig cells. In this study, we explored whether AQ affects steroidogenesis and cholesterol synthesis in Leydig cells.

## Materials and methods

### Reagents

AQ hydrochloride with 95% purity was purchased from Sigma-Aldrich (St. Louis, MO). Antibody against HMGCR, NR4A1, and SF-1 was obtained from Santa Cruz Biotechnology (Santa Cruz, CA) and used for immunoblot analysis and immunofluorescence staining.

### Cell culture

Mouse Leydig cell TM3 cells were obtained from American Type Culture Collection (Manassas, VA) and routinely maintained in a 1:1 mixture of Ham's F12 medium and DMEM supplemented with 2.5 mM l-glutamine, 15 mM Hepes, and 10% FBS (Thermo Fisher Scientific, Carlsbad, CA). Mouse primary Leydig cells were harvested from the testes of male mice (8–12 weeks old) as previously described (21). C57BL6/J male mice were maintained under specific pathogen-free conditions in accordance with the international guidelines approved by the Ewha Womans University's Institutional Animal Care and Use Committee (IACUC 17-013). Mouse testes were decapsulated and incubated with collagenase A (0.25 mg/ml) in a shaking water bath at 37°C for 15 min, and mouse Leydig cells were purified by discontinuous Percoll gradients of single-cell suspensions. Cells were resuspended in DMEM/F-12 and filtrated through a nylon mesh (100 μm pore), followed by plating with 2 × 10^6^/ml cell density. Cells were refreshed every day and cultured for 3 days.

### Cell viability assay

TM3 cells (1 × 10^4^/well) were plated in a 96-well plate and incubated with AQ for 24 h. Cells were subsequently incubated with Cellomax reagent for 2 h in the dark and subjected to a cytotoxicity assay using a Cellomax cell viability assay kit (Precaregene, Hanam, Gyeonggi-do, Korea). The absorbance was measured at 450 nm with a microplate reader (Molecular Devices, San Jose, CA) equipped at Drug Development Research Core Center. Separately, cells were stained with 3 μM of propidium iodide (Thermo Fisher Scientific) for 15 min at room temperature, followed by flow cytometry analysis and CellQuest quantitation.

### Quantitative real-time PCR

Total RNA was prepared from TM3 or primary Leydig cells using TRIzol reagent (Thermo Fisher Scientific) according to the manufacturer's protocol and reversely transcribed to complementary DNA. Quantitative real-time PCR was performed with THUNDERBIRD SYBR qPCR Mix (TOYOBO, Osaka, Japan) using a Step One Plus Real Time PCR system (Applied Biosystems, Foster City, CA). Relative transcript level was calculated after normalization with the Ct values of the *β-actin* gene. The specific primer sets were used: actin, 5′-caccctgtgctgctcaccgag-3′, 5′-accgctcgttgccaatagtga-3′; *ACC1*, 5′-aggacctggtggagtggctgga-3′, 5′-ctcctcctacgtggaagggg-3′; *CYP11A1*, 5′-aggactttccctgcgctcag-3′, 5′-atcgacgcatccttggggtcc-3′; *CYP17A1*, 5′-gcccaagtcaaagacacctaat-3′, 5′-gtacccaggcgaagagaataga-3′; *diacylglycerol acyltransferase* (*DGAT*), 5′-tcttaaagctggcggtcccc-3′, 5′-gacctgagccatcatggctg-3′; *FASN*, 5′-agtgtccaccaacaagcgccc-3′, 5′-aggagtgcccaatgatgcc-3′; *glycerol-3-phosphate* *acyltransferase* (*GPAT*), 5′-cttgcagaacagcagtggg-3′, 5′-gttcctttccgtcctggtg-3′; *HMGCR*, 5′-acagaggctgcagagcc-3′, 5′-agcagtgctttctccgtacc-3′; *lysophosphatidic acid acyltransferase* (*LPAAT*), 5′-accaccagagttccctcgacc-3′, 5′-tggggatgatggggacctggg-3′; *phosphatidic acid phosphatase* (*PAP*), 5′-acagttggactcattcaggg-3′, 5′-agcgcccctgtacccttcaggg-3′; *StAR*, 5′-tgtcaaggagatcaaggtcctg-3′, 5′-cgataggacctggttgatgat-3′; and *3βHSD*, 5′-ttgaccatgcctgggtggag-3′, 5′-tgrctccttccaacactgtc-3′.

### Testosterone and cholesterol ELISA

Testosterone and cholesterol production by Leydig cells was assay using a competitive Testosterone ELISA kit (Cayman Chemical, Ann Arbor, MI) and colorimetric cholesterol (mouse) ELISA kit (ECCH-100; BioAssay Systems, Hayward, CA) according to the manufacturer's instruction. For measuring testosterone, the cell culture supernatants and cell extracts were harvested and incubated with antibody against testosterone and secondary antibody bound to the assay plate. The plate was washed and incubated with Ellman′s reagent, and the absorbance was measured with a microplate reader. The testosterone ELISA kit has an assay range of 3.9–500 pg/ml and a sensitivity of approximately 6 pg/ml. For cholesterol measurement, the culture supernatants and cell extracts were incubated with anticholesterol antibody-coated plate and subsequently incubated with the secondary antibody conjugated with alkaline phosphatase. After incubation with the substrate, the absorbance was determined. The cholesterol ELISA kit has a sensitivity of 5 mg/dl. Clarify cell supernatant by centrifugation and collect the clarified supernatant. Cell extracts were harvested in extraction buffer containing 10 mM Hepes (pH 7.4), 150 mM NaCl, 1 mM EGTA, 0.1 mM MgCl_2_, and 0.5% Triton X-100.

### Immunoblot analysis

TM3 cells were treated with AQ, and protein extracts were harvested in RIPA lysis buffer. Protein extracts were resolved and blotted with anti-HMGCR antibody and subsequently incubated with antiactin antibody after stripping. Human embryonic kidney 293T (HEK293T) cells were transiently transfected with a NR4A1 expression vector, and protein extracts were subjected to immunoblot analysis with anti-NR4A1 antibody.

### Reporter gene assay

HEK293T or TM3 cells were transfected with the HMGCR-luc reporter gene and/or NR4A1 expression vector through a calcium phosphate transfection method and incubated with AQ for 24 h. Cell extracts were harvested with a reporter lysis buffer and assayed using a Luciferase assay kit (Thermo Fisher Scientific) and Galacto-Light Plus™ beta-Galactosidase Reporter Gene Assay System (Applied Biosystems). The RSVβ gene was also transfected for the normalization of transfection efficiency. Relative luciferase activity was determined after normalization with β-galactosidase activity.

### Immunofluorescence staining

TM3 cells were treated with or without AQ and fixed for 10 min with 4% formaldehyde in PBS, followed by permeabilization in 0.1% Triton X-100. After washing, cells were blocked for 30 min in a blocking buffer containing 3% BSA and 0.1% Tween-20 in PBS and incubated with antibody against NR4A1 and SF-1 overnight. Cells were washed and subsequently incubated with Alexa Fluor 488 or 647 conjugated secondary antibody for 1 h. Nuclei were counterstained using 4′,6-diamidino-2-phenylindole (Thermo Fisher Scientific). Cells were observed under a confocal microscopy (Zeiss LSM 880 Airyscan) at Ewha Imaging Core Center.

### BODIPY staining

TM3 cells were incubated with AQ for 24 h and washed with PBS. Intracellular lipid accumulation was visualized and quantitated by BODIPY staining. Cells were subsequently incubated with the non BODIPY™ 493/503 (2 μM; Thermo Fisher Scientific) fluorophore in the dark for 15 min at 37°C. Cells were washed with PBS three times and observed under a microscope. Quantitative analysis of BODIPY staining was conducted with an ImageJ software (National Institues of Health).

### DNA pulldown assay

HEK293T cells were transfected with mock or NR4A1 expression vector and subsequently treated with AQ for 24 h. Cells were resuspended and lysed in HKMG buffer (10 mM Hepes, pH 7.9, 100 mM KCl, 5 mM MgCl_2_, 10% glycerol, 1 mM DTT, and 0.1% NP-40) and incubated with 1 μg of biotin-labeled double-stranded DNA of the NBRE of the HMGCR gene promoter at 4°C overnight. The protein-DNA complex was precipitated with streptavidin-agarose beads and then subjected to SDS-PAGE and immunoblot analysis (23). Biotinylated single-stranded oligomers of NBRE within the HMGCR gene promoter were synthesized and annealed for DNA pulldown assay. The sequences are as follows: biotin-pHMGCR-NBRE-Top, 5′-ggcaagaccctgcaggtcaaactctga-3′ and pHMGCR-NBRE-bottom, 5′-ctcagagtttgacctgcagggtcttgc-3′.

### Lipidomics analysis using LC/MS

TM3 cells were incubated with vehicle or AQ (10 μM) for 24 h and subjected to high-throughput lipidomics analysis using ultra performance liquid chromatography coupled with quadrupole-TOF MS (1290 Infinity II LC Systems, Agilent, Santa Clara, CA). Cellular lipids were extracted with methyl *tert*-butyl ether and resuspended in 9:1 methanol:toluene and separated using an Acquity ultra performance liquid chromatography CSH C18 column (100 × 2.1 mm, 1.7 μm; Waters, MA) maintained at 65°C, and with flow rate of 0.6 ml/min. Mobile phases were 60:40 acetonitrile:H_2_O (A) and 90:10 isopropanol:acetonitrile (B) with 0.1% formic acid and 10 mM ammonium formate (24). The separation was conducted under the following gradient: 0 min 30% B; 0–2 min 30% B; 2–2.5 min 48% B; 2.5–11 min 82% B; 11–11.5 min 99% B; 11.5–12 min 99% B; 12–13 min 15% B; and 13–16 min 15% B. Quadrapule-TOF MS instrument was operated using the following parameters: gas flow, 8 l/min; gas temperature, 325°C; nebulizer, 35 psi; sheath gas flow, 11 l/min; sheath gas temperature, 350°C; and mass range, 300–1,200 *m/z*. The LC/MS data were analyzed by MS-DIAL, version 4.38, using an enriched LipidBlast library (25). Principal component analysis plot and heatmap from lipidomics data were performed using MetaboAnalyst 4.0 (https://www.metaboanalyst.ca/). All lipids were quantitatively evaluated by comparison with internal standards (e.g., 17:0 lysophosphatidylcholine, 10:0 phosphatidylcholine (PC), 10:0 phosphatidylethanolamine (PE), 17:0 SM (d18:1/17:0), and 17:0-17:1-17:0 D5 TG).

### Statistical analysis

Statistical analysis was performed with GraphPad Prism (GraphPad Software, San Diego, CA). All experiments were conducted at least three times, and data are expressed as the mean ± SEM. The statistical significance of differences among groups was analyzed by one-way ANOVA followed by Tukey's honest significant difference post hoc test or two-tailed Student's *t*-test. In all analyses, *P* values less than 0.05 were considered to indicate a statistically significant difference.

## Results

### AQ promotes expression of steroidogenic enzymes and increases testosterone production by Leydig cells

**Fig. 1:**
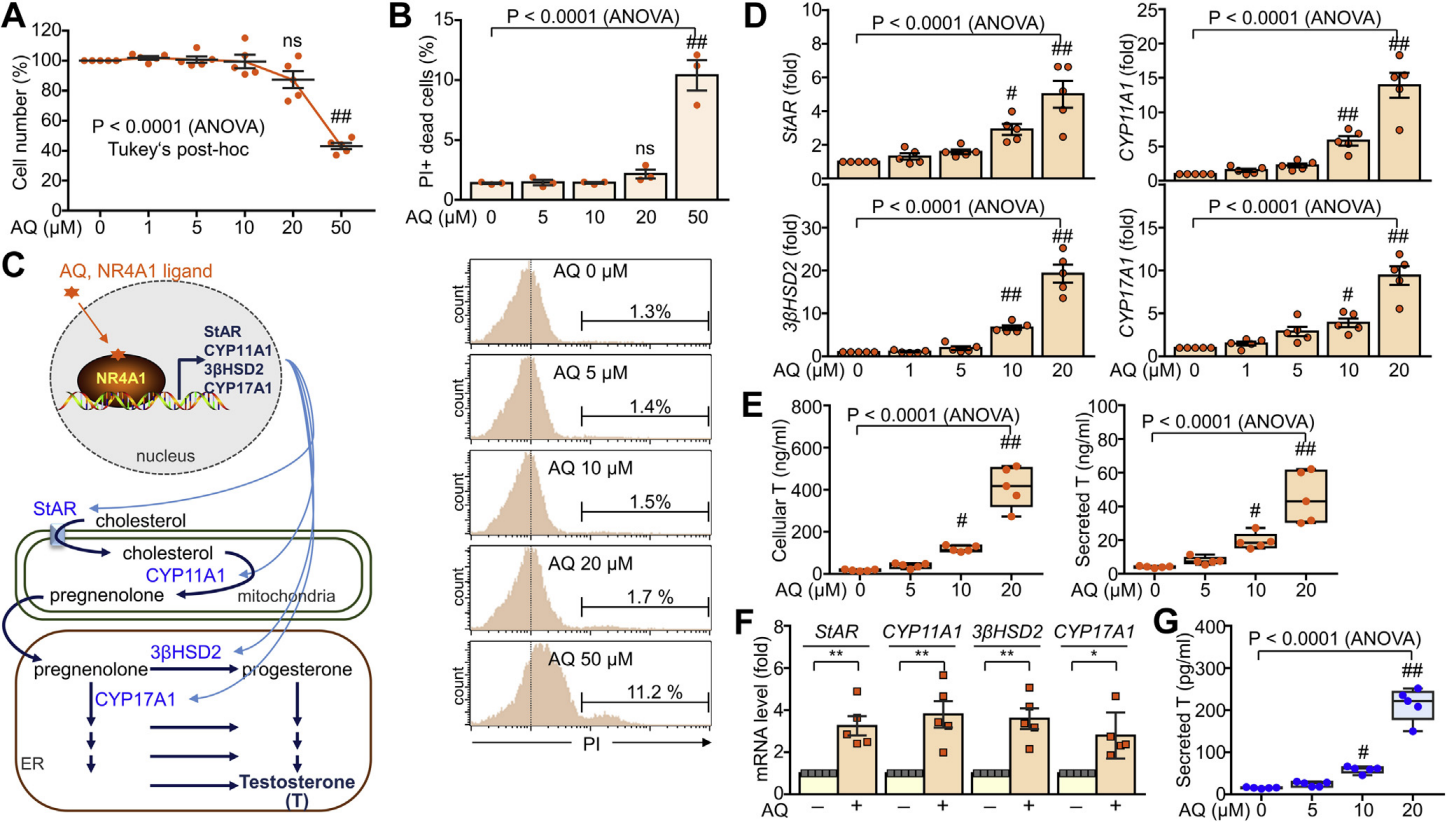
Increased testosterone synthesis in Leydig cells by AQ. TM3 cells were treated with AQ for 24 h and subjected to further analysis. A: The effect of AQ on cell viability was expressed in percentage change in cell number. B: Quantitative analysis of dead cells. C: Steroidogenesis in Leydig cells. NR4A1-induced steroidogenic enzymes convert cholesterol to testosterone (T) through activation of steroidogenic enzymes in mitochondria and endoplasmic reticulum (ER) of Leydig cells. D: Quantitative analysis of transcript levels of *StAR*, *CYP11A1*, *3βHSD*2, and *CYP17A1* in Leydig cells after treatment with AQ. E: Total cellular testosterone and secreted testosterone were determined in AQ-treated Leydig cells by ELISA. F: Relative transcript levels of steroidogenic enzymes were determined in primary Leydig cells that were treated with AQ (10 μM) for 24 h. G: Secreted testosterone was determined in primary Leydig cells after treatment with AQ. Data in A, B, D, E, F, and G are expressed as the mean ± SEM, and statistical analysis was conducted by Student′s *t*-test (A, F) or ANOVA with Tukey's honest significant difference post hoc test (D, E, and G). ∗*P* < 0.05; ∗∗*P* < 0.005; ∗∗∗*P* < 0.0005 by Student's *t*-test. ^#^*P* < 0.05; ^##^*P* < 0.01 compared with control (AQ = 0 μM) by Tukey's post hoc test. ns, not significant.

To evaluate the effects of AQ on steroidogenesis in Leydig cells, we first examined the effects of different concentrations of AQ on the viability of TM3 Leydig cells. AQ decreased the cell viability of Leydig cells by 60% at 50 μM but had no significant cytotoxicity at concentrations below 20 μM. AQ appeared to reduce cell viability by approximately 15% at 20 μM, but it was a statistically insignificant decrease compared with the control (Fig. 1A). Further analysis of dead cells by propidium iodide staining confirmed a significant increase in cell death only in 50 μM of AQ (Fig. 1B). Since *StAR*, *CYP11A1*, *3β-HSD2*, and *CYP17A1* are NR4A1 target genes involved in steroidogenesis, we next examined their expression levels after treatment with AQ (Fig. 1C). As expected, AQ significantly increased the transcript levels of *StAR*, *CYP11A1*, *3βHSD2*, and *CYP17A1* in TM3 Leydig cells in a dose-dependent manner (Fig. 1D). Consistent with these results, the total cellular testosterone level increased after treatment with AQ, and secreted testosterone also increased with increasing AQ concentrations (Fig. 1E). Furthermore, the expression of steroidogenic enzymes and testosterone production was significantly increased in primary Leydig cells after AQ treatment (Fig. 1F, G).

### AQ increases cholesterol production by Leydig cells

**Fig. 2:**
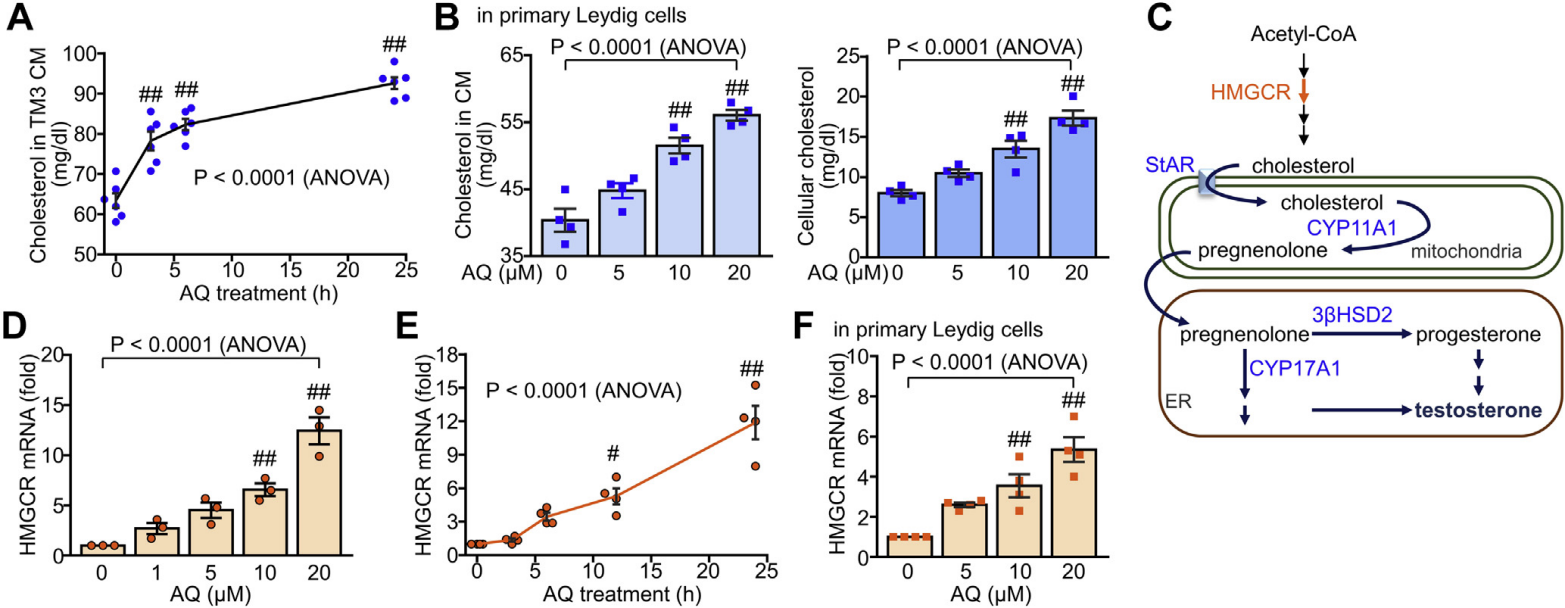
Enhanced cholesterol biosynthesis in Leydig cells by AQ. A: TM3 cells were incubated with AQ (10 μM) for 24 h, and the culture supernatant was used to determine cholesterol by ELISA. B: Primary Leydig cells were incubated with AQ for 24 h, and secreted and cellular cholesterol levels were determined by ELISA. C: Cholesterol biosynthesis and steroidogenesis. D, E: TM3 cells were treated with different concentration of AQ (D) and 10 μM AQ for various times (E), and relative transcript level of HMGCR was determined by quantitative real-time PCR analysis. F: The relative transcript level of *HMGCR* was determined in primary Leydig cells that were treated with AQ. Data in A, B, D, E, and F are expressed as the mean ± SEM, and statistical analysis was conducted by ANOVA with Tukey's honest significant difference post hoc test. ^#^*P* < 0.05; ^##^*P* < 0.01 compared with control (AQ = 0 h or 0 μM) by Tukey's post hoc test.

Since cholesterol is a precursor for testosterone biogenesis, we attempted to determine whether AQ affects cholesterol production by Leydig cells. Interestingly, ELISA with cell supernatants demonstrated that AQ treatment significantly elevated the extracellular cholesterol level in TM3 cells 3 h after treatment (Fig. 2A). In addition, both extracellular and intracellular levels of cholesterol were increased in primary Leydig cells following treatment with AQ (Fig. 2B). Since cholesterol can be synthesized by cholesterol biogenesis enzymes, such as HMGCR, the effects of AQ on HMGCR gene transcription were assessed in Leydig cells (Fig. 2C). *HMGCR* transcript levels were increased by AQ treatment in dose-dependent and time-dependent manners (Fig. 2D, E). The increased expression of *HMGCR* induced by AQ was also confirmed in primary Leydig cells (Fig. 2F). These results suggest that AQ promotes cholesterol synthesis through the induction of *HMGCR* gene transcription, leading to increased testosterone biogenesis.

### AQ enhances HMGCR expression through induction of nuclear expression of NR4A1

**Fig. 3:**
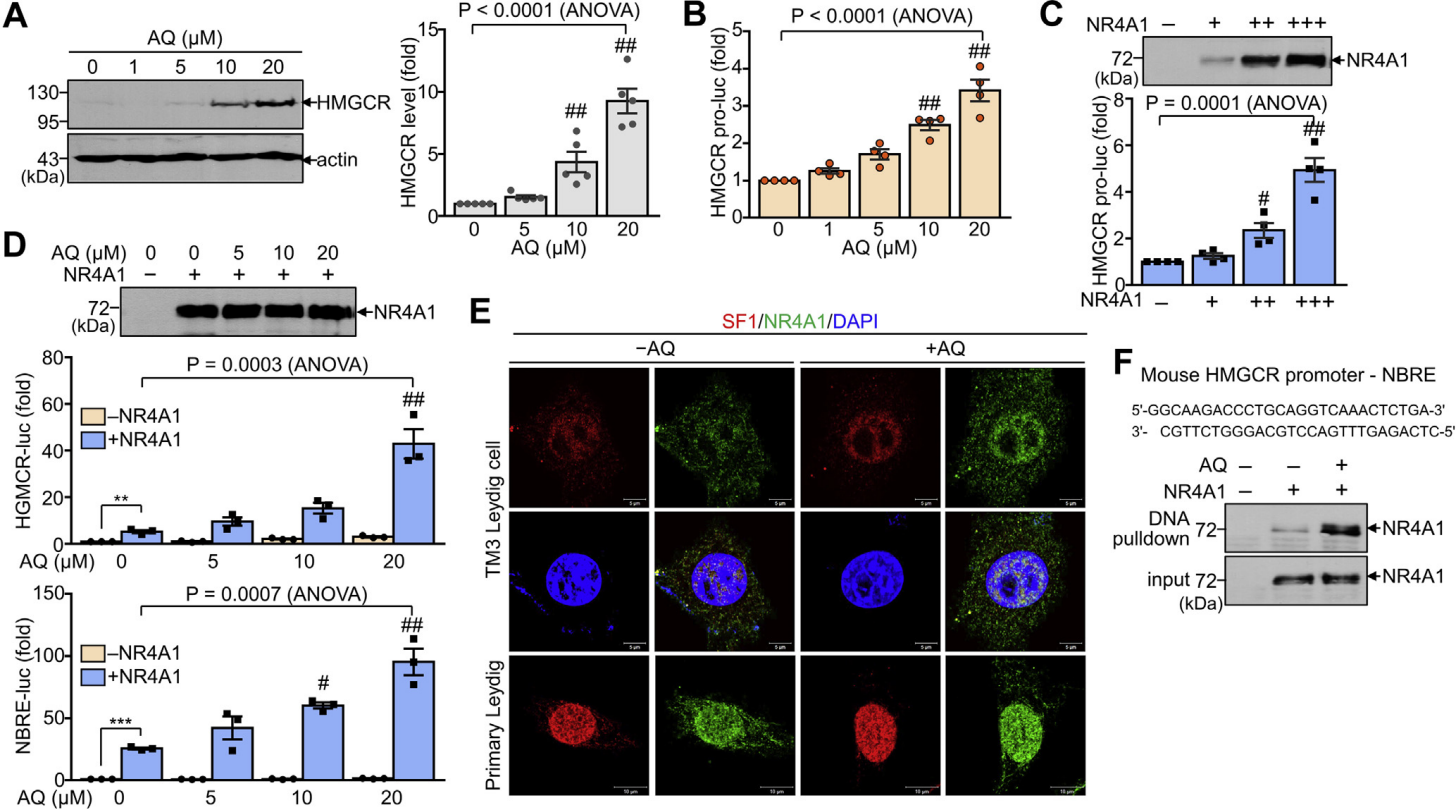
Increased expression of HMGCR by AQ in Leydig cells. A: TM3 cells were incubated with AQ with different concentration of AQ for 24 h. Cell extracts were analyzed by immunoblot with anti-HMGCR antibody. Protein band intensities were quantitated from five independent blots using ImageJ software. B–D: TM3 cells were transfected with HMGCR-luc with or without NR4A1 and subsequently incubated with AQ for 24 h. B: The effect of AQ on HMGCR-luc reporter activity was calculated in TM3 cells. C: NR4A1 effect on HMGCR-luc reporter activity was determined after normalization with β-galactosidase activity. D: Effects of NR4A1 and AQ on HMGCR-luc or NBRE-luc reporter activity were determined in TM3 cells. NR4A1 expression was analyzed by immunoblot analysis in HEK293T cells. E: TM3 cells and primary Leydig cells were treated with either vehicle or AQ and stained with antibody against NR4A1 and SF-1. Cells were also stained with DAPI. Representative image out of five independent experiments is shown. F: DNA pulldown assay. NRA1 was overexpressed in HEK293T cells, which were treated with or without AQ. Cell extracts were incubated with the NBRE DNA within the HMGCR gene promoter and analyzed by immunoblot analysis with anti-NR4A1 antibody. Data in A–D are expressed as the mean ± SEM, and statistical analysis was conducted by Student′s *t*-test or ANOVA with Tukey's honest significant difference post hoc test. ∗∗*P* < 0.005; ∗∗∗*P* < 0.0005 by Student's *t*-test. ^#^*P* < 0.05; ^##^*P* < 0.01 compared with control (AQ = 0 μM) by Tukey's post hoc test. DAPI, 4′,6-diamidino-2-phenylindole.

As AQ increased the expression of HMGCR in Leydig cells, we attempted to confirm the effect of AQ on HMGCR expression and clarify the molecular relationship between NR4A1 and its gene expression (Fig. 3A). Consistent with the increased transcript levels of HMGCR, AQ dose-dependently increased the protein expression of HMGCR (Fig. 3A). Therefore, we established a reporter gene containing the HMGCR gene promoter and assessed whether the HMGCR reporter activity was affected by AQ or NR4A1. As shown by the increased transcript levels of *HMGCR* by AQ treatment, AQ dose-dependently promoted *HMGCR* promoter activity (Fig. 3B). In addition, ectopic NR4A1 expression significantly enhanced *HMGCR* promoter activity, whereas increased expression of ectopic NR4A1 was confirmed in HEK293T cells (Fig. 3B, C). As previously reported (22), NR4A1 overexpression increased the NBRE reporter activity that contains four copies of NR4A1-binding elements, which was further increased by AQ treatment. Consistently, ectopic overexpression of NR4A1 significantly increased *HMGCR* promoter activity and further enhanced in the presence of AQ. And the NR4A1 expression level was not altered by AQ treatment (Fig. 3D). More interestingly, AQ increased the nuclear expression of NR4A1 in TM3 and primary Leydig cells, whereas nuclear SF-1 expression was not affected by AQ (Fig. 3E). In addition, AQ further potentiated the DNA-binding activity of NR4A1, as evidenced by the increased complex formation of NR4A1 with NBRE DNA within the HMGCR gene promoter (Fig. 3F). These results indicate that AQ increases NR4A1-mediated gene transcription of HMGCR through the induction of nuclear NR4A1 expression, resulting in cholesterol biogenesis.

### AQ increases lipid accumulation in Leydig cells through induction of fatty acid synthesis

**Fig. 4:**
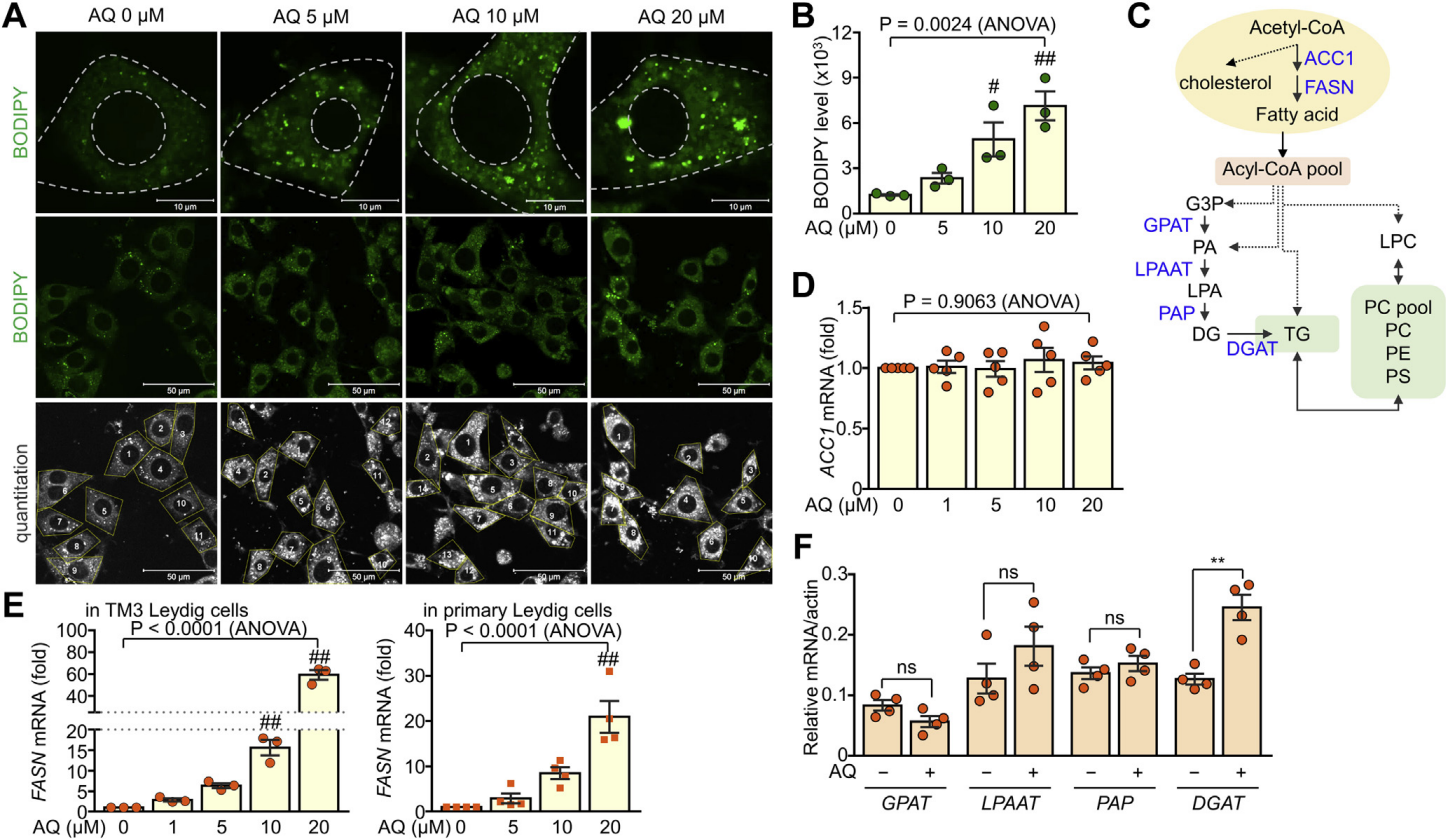
Increased lipid accumulation in AQ-treated Leydig cells. A: TM3 cells were treated with AQ and subjected to BODIPY staining. B: Quantitation of BODIPY staining intensity. C: The process for fatty acid synthesis and lipid biogenesis. D: TM3 cells were incubated with AQ, and relative transcript level of *ACC1* was determined after normalization with actin level. E: TM3 cells and primary Leydig cells were treated with AQ for 24 h, and relative transcript level of *FASN* was determined by quantitative real-time PCR analysis. F: The relative transcript levels of lipogenic genes were determined in TM3 Leydig cells. Data in B, D, E, and F are expressed as the mean ± SEM. Statistical analysis was conducted by ANOVA with Tukey's honest significant difference post hoc test (B, D, and E) or Student's *t*-test (F). ^#^*P* < 0.05; ^##^*P* < 0.01 compared with control (AQ = 0 μM) by Tukey's post hoc test. ∗∗*P* < 0.005 by Student's *t* test. ns, not significant.

Consistent with the increase in testosterone and cholesterol biosynthesis by AQ treatment, intracellular lipid accumulation in Leydig cells was increased by AQ, as evidenced by BODIPY staining (Fig. 4A). Quantitative analysis also confirmed that AQ significantly enhanced lipid accumulation in Leydig cells (Fig. 4B). Abundant intracellular acetyl-CoA levels essential for cholesterol synthesis may increase cholesterol biosynthesis as well as fatty acid synthesis. The concomitant increase in acyl-CoA pool not only induces conversion to structural lipids such as lysophosphatidylcholine, PC, PE, and phosphatidylserine but also increases the conversion to TG by the action of *GPAT*, *LPAAT*, *PAP*, and *DGAT* (16, 26) (Fig. 4C). Therefore, we also analyzed the effect of AQ on fatty acid synthesis and subsequent storage lipid conversion because of accumulated lipid vesicles. Although *ACC1* expression was not changed by AQ treatment, FASN was prominently increased by AQ at the transcriptional level in both TM3 and primary Leydig cells (Fig. 4D, E). Furthermore, the lipid-modifying enzymes *GPAT*, *LPAAT*, and *PAP* were not affected by AQ, whereas *DGAT* was significantly increased by AQ in Leydig cells (Fig. 4F). These results indicate that AQ significantly increased lipid biogenesis, particularly fatty acids and storage lipid TG, resulting in accumulation of lipid vesicles.

### AQ changes cellular lipid composition and enhances TG accumulation in Leydig cells

**Fig. 5:**
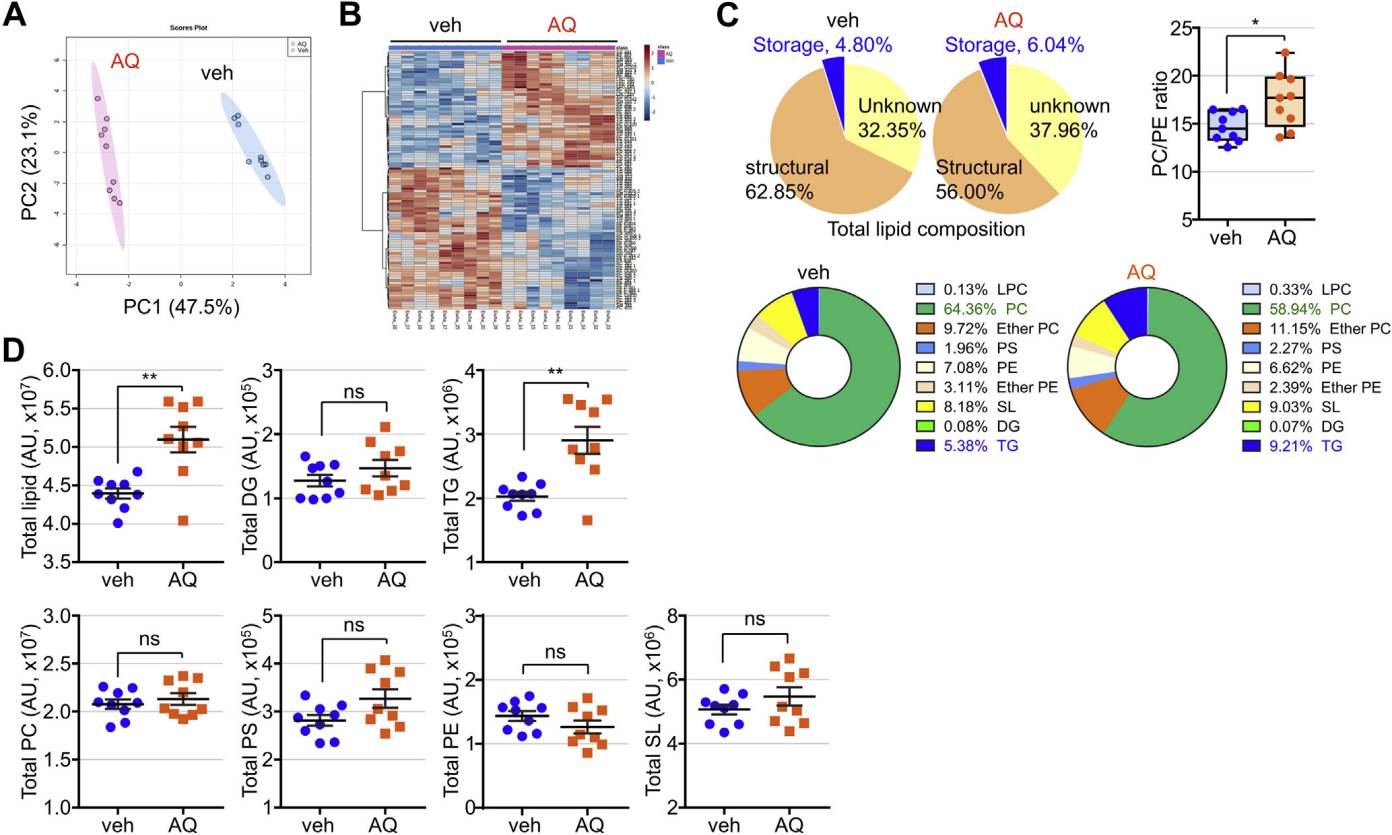
Alterations in lipid composition and increased TG synthesis in response to AQ. TM3 cells were incubated with AQ for 24 h, and cell extracts were subjected to lipidomics analysis. A: The PCA scores 2D plot of LC/MS-based lipid profiles from vehicle- or AQ-treated TM3 cells. B: The heatmap of lipid profile expression in TM3 cells treated with vehicle or AQ. C: Proportions of the identified lipids as well as unknown lipids by LC/MS based on lipid analysis. The ratio of cellular PC/PE was determined in vehicle- or AQ-treated Leydig cells. D: Relative intensity of lipids was determined in vehicle- and AQ-treated TM3 cells. Data in C, D are expressed as the mean ± SEM (*n* = 9). ∗*P* < 0.05; ∗∗*P* < 0.005 by Student's *t*-test. ns, not significant; PCA, principal component analysis.

Since AQ increases lipid accumulation in Leydig cells, we attempted to analyze cellular lipid composition using a lipidomics approach. Principal component analysis plot revealed that AQ distinctively changed the cellular lipid composition of Leydig cells (Fig. 5A). Extensive changes in lipid composition were observed in Leydig cells after treatment with AQ, as visualized by a heatmap (Fig. 5B). LC/MS-based lipid analysis confirmed that 67.3 and 62.0% of total lipids were identified in vehicle- and AQ-treated Leydig cells, respectively, but AQ decreased structural lipids and increased storage lipids (Fig. 5C). The most abundant structural lipids, PCs, were decreased in proportion in AQ-treated cells, whereas the percentage of the TG storage lipid was significantly increased by AQ treatment. The ratio of PC:PE was slightly but significantly increased in AQ-treated Leydig cells, reflecting adequate membrane integrity and cell viability (27). Further quantitative analysis showed that the overall amount of total lipids was significantly increased in Leydig cells after AQ treatment, showing the same quantitative level of structural lipids despite the lower proportion (Fig. 5D). Interestingly, the amount of intracellular TG was significantly elevated in Leydig cells after treatment with AQ, which was also consistent with an increase in the proportion of TG in total lipids in AQ-treated cells.

## Discussion

The antimalarial drug AQ not only significantly increased the expression of steroidogenic enzymes and testosterone production by Leydig cells in the absence of LH/LHR signaling but also potently enhanced cholesterol biosynthesis through the induction of NR4A1-mediated *HMGCR* expression. AQ promoted nuclear expression of NR4A1 in Leydig cells, resulting in a significant increase in the transcriptional and DNA-binding activities of NR4A1. Furthermore, AQ elevated total intracellular lipids in Leydig cells and promoted TG accumulation via the induction of *FASN* and *DGAT* transcription.

The key steroidogenic enzymes StAR and CYP11A1 are mainly regulated by SF-1 at the transcriptional level (28, 29, 30, 31). The proximal and distal regions of the *StAR* and *CYP11A1* gene promoters interact with SF-1 to efficiently induce gene transcription (29, 30). Thus, SF-1 deficiency reduces testosterone production by Leydig cells, as in StAR or CYP11A1 deficiency. Failure to produce testosterone because of deficiency of SF-1, StAR, or CYP11A1 leads to a marked accumulation of TG and cholesterol concomitantly with failure to consume cholesterol (19). In addition to SF-1, NR4A1 has also been suggested as a transcriptional activator of *StAR*, *CYP11A1*, *CYP17A1*, and *HSD3β genes* (21, 32). Although both SF-1 and NR4A1 are important for inducing steroidogenic genes, it is unclear which signaling modulates the activity of SF-1 and NR4A1, respectively, and whether SF-1 and NR4A1 cooperatively regulate steroidogenic gene transcription (33). In this study, AQ selectively induced NR4A1 activity and increased the expression of NR4A1-mediated steroidogenic enzymes. We also confirmed that NR4A1 increased the expression of *HMGCR* and that AQ further potentiated NR4A1-mediated *HMGCR* expression, resulting in the accumulation of cholesterol. AQ-induced cholesterol accumulation is due to an increase in HMGCR expression, which is distinct from cholesterol accumulation resulting from failure to consume cholesterol in SF-1, StAR, and CYP11A1 deficiency. Since AQ increased the expression of *FASN* and *DGAT*, NR4A1 may also be important for the transcriptional regulation of FASN and DGAT through binding to their gene promoters. Furthermore, increased fatty acid synthesis and TG accumulation by AQ may also have the benefit of providing free fatty acids to convert cholesterol to cholesteryl ester to store precursors of testosterone synthesis (26) and generating sufficient energy for cholesterol biosynthesis via β-oxidation (34). Therefore, AQ may efficiently produce testosterone in Leydig cells even in the absence of LH/LHR signaling through the de novo synthesis of cholesterol, fatty acids, and TG.

Testosterone production by Leydig cells is critical for testicular spermatogenesis and maintenance of male fertility and is highly dependent on cholesterol homeostasis (35, 36). Since Leydig cells take plasma cholesterol, which is synthesized primarily in the liver, and produce testosterone (37), the depletion of cholesterol in plasma and tissues leads to male infertility (38). Lipid-lowering drugs such as statins inhibit testosterone production by Leydig cells (39). In addition, abnormally elevated plasma cholesterol levels in patients with hyperlipidemia or metabolic syndrome may also affect reproductive function and cause male infertility (40). The prevalence of testosterone deficiency is closely related to aging and common medical conditions, including obesity, diabetes, and hypertension (41, 42). Testosterone therapy has potential benefits of improved reproductive function, improved mood and well-being, and increased muscle mass and bone density. However, testosterone may increase the risk of cardiovascular complications, prostate cancer development, polycythemia, and venous thromboembolism (43). Therefore, it is necessary to develop therapeutics that can replace testosterone therapy. Since treatment with AQ increased both cholesterol and testosterone biosynthesis in Leydig cells, it can also be a beneficial therapeutic for treating testosterone deficiency because of its steroidogenic activity. AQ also improves insulin resistance and lipid metabolism in diabetic model mice by activating PPARα/γ and thus can be useful in preventing and treating type 2 diabetes. Accordingly, testosterone deficiency with type 2 diabetes may benefit from the administration with AQ (44, 45). However, it is still to be confirmed whether AQ affects cholesterol synthesis in hepatocytes other than Leydig cells and whether AQ alters the blood levels of cholesterol and testosterone following the clinical application.

## Data availability

The authors confirm that the data supporting the findings of this study are available within the article and its supplemental data.

## Supplemental data

This article contains supplemental data.

## Conflict of interest

The authors declare that they have no conflicts of interest with the contents of this article.
